# Biomedical Applications of Computer Vision Using Artificial Intelligence

**DOI:** 10.1155/2022/9843574

**Published:** 2022-01-26

**Authors:** Vahid Rakhshan, Alexandre Hideki Okano, Zhiyong Huang, Gianluca Castelnuovo, Abrahão F. Baptista

**Affiliations:** ^1^Department of Cognitive Neuroscience, Institute for Cognitive Science Studies, Tehran, Iran; ^2^Center for Mathematics, Computation and Cognition, Universidade Federal do ABC (UFABC), São Bernardo do Campo, Santo Andre 09606-045, Brazil; ^3^Department of Computer Science, NUS School of Computing, National University of Singapore, Singapore; ^4^Department of Psychology, Catholic University of Milan, Mila, Italy; ^5^Istituto Auxologico Italiano IRCCS, Psychology Research Laboratory, Milan, Italy

Computational neuroscience is concerned with simulating real neural systems to predict brain workings and disorders from subneuronal systems to network plasticity as hypotheses to be tested later in real neural tissues and hence to understand the principles governing them. Some ideas from this field can be used in artificial intelligence and other fields. Mimicking the central nervous system and by extension and creating various additional methods of computation such as artificial neural networks, machine learning, deep learning, or genetic algorithms have led to the artificial intelligence field which aims to solve given problems in a flexible, intelligent, and learnable way. The advent of these fields has numerous biomedical applications such as image processing and computer vision, machine learning, and deep learning for the assessment of imaging and signal datasets, disease diagnostic systems, expert systems to offer and optimize treatment planning, brain-computer interface, smart prosthetic limbs, and many others.

Image processing is a subfield of digital signal processing and a vast set of techniques used to enhance or manipulate digital images in order to make them more practical in different ways for different purposes. Computer vision is a field of computer science concerned with “understanding” images, videos, or 3D volumes by the computer through extracting desired features and attributes of images by various sets of algorithms and techniques.

This issue sought to publish select original and review articles on clinical and paraclinical applications of artificial intelligence and computational neuroscience in computer vision such as structural and functional brain imaging, histopathology, microbiology, surgery, and medical and dental radiography/tomography.

It published 6 articles: “Estimating Gender and Age from Brain Structural MRI of Children and Adolescents: A 3D Convolutional Neural Network Multitask Learning Model” by Mendes et al; “Denoising of 3D Brain MR Images with Parallel Residual Learning of Convolutional Neural Network Using Global and Local Feature Extraction” by Wu et al; “SGPNet: A Three-Dimensional Multitask Residual Framework for Segmentation and IDH Genotype Prediction of Gliomas” by Wang et al; “Classification of Hematoxylin and Eosin-Stained Breast Cancer Histology Microscopy Images Using Transfer Learning with EfficientNets” by Munien and Viriri; “A Semisupervised Learning Scheme with Self-Paced Learning for Classifying Breast Cancer Histopathological Images” by Asare et al; and “A Novel Bayesian Approach for EEG Source Localization” by Oikonomou and Kompatsiaris.

*Vahid Rakhshan* *Alexandre Hideki Okano* *Zhiyong Huang* *Gianluca Castelnuovo* *Abrahão F. Baptista*

## Data Availability

There are no data associated with this paper.

